# Study on the Mechanism by Which Fe^3+^ Promotes Toluene Degradation by *Rhodococcus* sp. TG-1

**DOI:** 10.3390/microorganisms13020468

**Published:** 2025-02-19

**Authors:** Yue Qiao, Jiajun Ma, Lei Huang, Guohui Gao, Yihe Zhao, Agostinho Antunes, Meitong Li

**Affiliations:** 1 Tianjin Key Laboratory of Organic Solar Cells and Photochemical Conversion, College of Chemistry and Chemical Engineering, Tianjin University of Technology, Tianjin 300384, China; qiaoyue@stud.tjut.edu.cn (Y.Q.); martintin@stud.tjut.edu.cn (J.M.); gaoguohui@stud.tjut.edu.cn (G.G.); 2 CIIMAR/CIMAR, Interdisciplinary Centre of Marine and Environmental Research, University of Porto, Terminal de Cruzeiros do Porto de Leixões, Av. General Norton de Matos, s/n, 4450-208 Porto, Portugal; yihe.zhao@ciimar.up.pt (Y.Z.); aantunes@ciimar.up.pt (A.A.); 3 Department of Biology, Faculty of Sciences, University of Porto, Rua do Campo Alegre, s/n, 4169-007 Porto, Portugal

**Keywords:** toluene biodegradation, Fe^3+^, *Rhodococcus* sp., transcriptome

## Abstract

Volatile organic compound pollution caused by toluene has become a global issue. In order to solve this problem, biodegradation of toluene has been applied all over the world. This study investigated the effects of Fe^3+^ on toluene degradation by the *Rhodococcus* sp. TG-1. The results show that 1 mg L^−1^ Fe^3+^ increased the degradation rate of 600 mg L^−1^ toluene from 61.9% to 87.2% at 16 h. The acceleration mechanism of Fe^3+^ was explicated using transmission electron microscope (TEM) and energy-dispersive X-ray spectroscopy (EDX) analyses, coupled plasma optical emission spectroscopy, an enzyme activity assay, and transcriptome analysis. Four genes were detected to be significantly up-regulated under Fe^3+^ induction, suggesting that Fe^3+^ might be implicated in toluene degradation. Meanwhile, Fe^3+^ was a component of the active center of catechol 1,2-dioxygenase (C12O) and significantly improved the enzyme activity of C12O. The mechanism by which Fe^3+^ accelerates toluene degradation was proposed based on the transcription levels of degradation genes and the enzyme activity of C12O. This study provided an improved method for enhancing the degradation effect of toluene and furthered our comprehension of the mechanism of toluene degradation.

## 1. Introduction

Toluene has been widely used in the production of medicine, rubber, glass, solar panels, and aerospace equipment and in mining operations. The widespread use of toluene increases the possibility of environmental pollution [1,2]. Toluene results in the formation of photochemical smog, ozone, and secondary organic aerosols when released into the air. Ozone and secondary organic aerosols are harmful to human health and hamper air quality [3,4]. Toluene is one of the priority pollutants for the Environmental Protection Agency (EPA) [5,6]. Hence, advanced technologies are imperative for the treatment of toluene. The common methods mainly include adsorption, regenerative thermal oxidation, regenerative catalytic oxidation, ultraviolet photocatalysis, and biological technologies [7]. Biological technologies present specific advantages when simultaneously removing toluene, such as low operating costs, no secondary pollution, and mild operating conditions. Bacteria can degrade toluene into a non-toxic substance, which eventually turns into CO_2_ and H_2_O [8]. The main limitations of toluene degradation by bacteria include a slow degradation rate and low transformation efficiency. Therefore, bacteria with high degradation activity and efficiency are essential for the further development of biological degradation processes.

Researchers have found that Fe^3+^ plays an active role in bacteria used for organic compound biodegradation. Recently, it has been pointed out that Fe^3+^ in minute quantities also has potential applications in toluene biodegradation, highlighting the need to extend our knowledge of the degradation mechanism of toluene. The main metabolites of toluene biodegradation are benzene, lipids with benzene rings, and diols. Catechol 1,2-dioxygenase (C12O) is a key enzyme involved in the degradation pathways of toluene. C12O catalyzes the cleaving activities of catechol, an intermediate product in the degradation of toluene. An Fe atom is the active center of C12O, and Fe^3+^ has a strong influence on the catalytic ability of C12O [9]. The Fe^3+^ in the active site of C12O coordinates with the two hydroxyl groups of catechol, then binds molecular oxygen to achieve the cleavage of catechol through electron transfer [10].

This study explained the mechanism by which Fe^3+^ enhances the toluene degradation efficiency of the *Rhodococcus* sp. TG-1. Therefore, two main parts of this study are discussed: (1) Fe^3+^ stimulated the activity of C12O, and (2) Fe^3+^ enhanced the expression of toluene-degrading genes.

## 2. Materials and Methods

### 2.1. Bacteria and Culture Media

The preparation of minimal salt media (MSM) and Luria–Bertani (LB) media was carried out according to previous studies [11]. Toluene (99.5% purity) was obtained from Tianjin bohua Chemical Agent Co., Ltd. (Tianjin, China).

A strain was isolated from oil-contaminated soil from the Liaohe Oil Field, China (41.13–41.26 N, 121.08–121.09 E). Soil samples were added to an MSM containing 600 mg L^−1^ toluene and cultured for 5 days. The next batch of medium was inoculated at a 2% volume ratio, and this enrichment process was repeated 5 times consecutively. After appropriate dilution, the bacterial suspension was spread on solid LB plates. All single colonies were then inoculated into an MSM containing 600 mg L^−1^ toluene and cultured for 5 days. The strain with the fastest growth rate and highest cell density was selected as the study strain. The isolated *Rhodococcus* sp. TG-1 strain was subjected to phylogenetic analysis.

### 2.2. Bacterial Identification, Genome Sequencing, and Annotation

Extraction and analysis of genomic DNA were carried out according to a previous study [12]. The raw data of the genome were submitted to the NCBI and can be accessed in the Sequence Read Archive (SRA) under BioProject PRJNA739011; BioSample SAMN19769069; and accessions CP077417, CP077418, CP077419, and CP077420. The strain accession numbers were retrieved from the GenBank database using the neighbor-joining method. MEGA 7.0 software was used to compare microorganisms with related sequences to identify the genus of these strains.

### 2.3. The Effects of Various Metal Ions on Toluene Degradation

Strain TG-1 was cultured in LB medium for 48 h, then harvested by centrifugation at 4000 rpm for 2 min. The cells were then washed with sterile water to discard residual organic compounds. The prepared bacteria were diluted to an OD_600_ of 2.0 ± 0.05 for inoculation. The bacterial culture (2%, *v*/*v*) was subsequently added to 30 mL of an MSM supplemented with 18 mg of toluene (600 mg L^−1^) in 250 mL Erlenmeyer flasks. The toluene concentration was measured by gas chromatography (GC) every 8 h. The flasks were sealed using gastight crimped Teflon-coated butyl rubber stoppers to prevent toluene leakage. The culture conditions were 200 rpm and 25 °C, with all samples prepared in triplicate.

Stock solutions of Fe^3+^, Mn^2+^, Sr^2+^, Zn^2+^, Cr^3+^, Pb^2+^, Cu^2+^, and Ni^2+^ were prepared using the following compounds: FeCl_3_·6H_2_O, MnCl_2_·4H_2_O, SrCl_2_·6H_2_O, ZnCl_2_, CrCl_3_·6H2O, PbCl_2_, CuCl_2_·2H_2_O, and NiCl_2_·6H_2_O. The final concentration of heavy metal ions in the stock solution was 10 g L^−1^. An MSM with metal ions that lacked bacterial cells served as the abiotic control, while an MSM with bacterial cells but without metal ions served as the normal control. All filter-sterilized metal ion salt stock solutions were prepared separately. The degradation percentage of toluene was measured at 16 h. The inoculation mode and culture conditions were the same as described above.

The toluene was taken as 1.0 mL gas samples from the headspaces of the experimental samples and controls (without strain TG-1) using a gastight 5 mL Gaoge syringe (Gaoge, Shanghai, China). A gas chromatographic system (BRUKER 456-GC, San Jose, CA, USA) was used to detect variations in toluene. The GC procedures and headspace method were performed as previously reported [13,14]. The degradation percentage of toluene was calculated as follows:

Degradation percentage = [(Initial toluene − Residual toluene)/Initial toluene] × 100%

### 2.4. Iron Ions’ Influence on Toluene Degradation

Preliminary preparation was the same as in Section 2.3. An MSM with toluene that lacked iron ions served as the control. TG-1 was cultured in 30 mL of an MSM with 600 mg L^−1^ toluene and 1 mg L^−1^ Fe^3+^ for 16 h, then harvested by centrifugation at 4000 rpm for 2 min. The supernatant and TG-1 cells were used for the following experiment.

#### 2.4.1. Extracellular and Intracellular Iron Contents

Strain TG-1 was cultured in an Fe^3+^-supplemented system and harvested. The concentration of Fe^3+^ in the supernatant was tested by coupled plasma optical emission spectroscopy (ICP-OES) after centrifugation. An MSM with Fe^3+^ that lacked bacterial cells served as the abiotic control. The intracellular Fe^3+^ concentration was obtained by subtracting the Fe^3+^ concentration in the supernatant from the Fe^3+^ concentration in the abiotic control.

#### 2.4.2. TEM and EDX Analysis

Transmission electron microscopy (TEM) analysis of strain TG-1 was carried out using a JEM1200EX transmission electron microscope (JEOL, Tokyo, Japan) equipped with an energy-dispersive X-ray spectroscope (EDX, FEI TalosF200X) (ThermoFisher, Waltham, MA, USA) operating at 200 kV. The TG-1 cells were diluted 10 times with deionized water, and 100 μL of this suspension was added dropwise on an ultrathin carbon-coated copper mesh and allowed to dry.

#### 2.4.3. Enzyme Activity Assay

The bacterial cells were harvested by centrifugation at 8000 rpm and 4 °C for 10 min. The harvested cells were washed twice with 30 mL of a 0.05 mM sodium phosphate buffer (pH = 7.0) and resuspended in the same buffer to prepare them for disruption (OD600 = 0.25). Thereafter, 20 mL of cell suspension was subjected to cell disruption at 150 W for 15 min (2 s: 4 s pulses on an on–off basis), and a crude enzyme solution was subsequently obtained by centrifugation at 10,000 rpm for 25 min at 4 °C. The reaction system contained 3 mL of the crude enzyme solution, 0.1 mmol L^−1^ catechol, and a 0.05 mol L^−1^ sodium phosphate buffer. The amount and activity of C12O were tested according to previous studies [15].

### 2.5. RNA Extraction, Transcriptome Assembly, and Analysis

TG-1 was cultured in 30 mL of an MSM with 600 mg L^−1^ toluene and 1 mg L^−1^ Fe^3+^ for 16 h; samples inoculated with TG-1 and toluene but without any metal ions (blank controls) were also included. Total RNA was extracted using an RNAprep Pure Cell/Bacteria Kit (Tiangen, Beijing, China) according to the manufacturer’s instructions. RNA-seq transcriptome libraries were constructed using a TruSeqTM RNA Sample Preparation Kit (Illumina, San Diego, CA, USA) and were then sequenced on an Illumina HiSeq X ten platform with a 2 × 150 bp read length. The differentially expressed genes (DEGs) were transmitted into GO (gene ontology) and KEGG (Kyoto Encyclopedia of Genes and Genomes) analyses. Genes with a *p*-value < 0.05 and |log2FC| > 1 were classified as differentially expressed.

### 2.6. Validation of RT-qPCR

cDNA was synthesized using 500 ng of RNA as a template with a TOYOBO qPCR Kit (TOYOBO, Osaka, Japan). RT-qPCR was performed using SYBR green fluorescent dye (GenStar, Beijing, China) on a two-color real-time PCR detection system (LightCycler^®^ 96, Roche, Basel, Switzerland). The experimental data were analyzed according to the 2^−ΔΔCt^ method and standardized using the 16S rRNA gene as an internal reference control [15,16]. The thermocycling conditions were as follows: initial denaturation at 95 °C for 3 min, followed by 40 cycles of 95 °C for 5 s and 60 °C for 30 s. The qPCR primers used are listed in Table 1.

## 3. Results and Discussion

### 3.1. Isolation and Characterization of Bacteria

**Figure 1:**
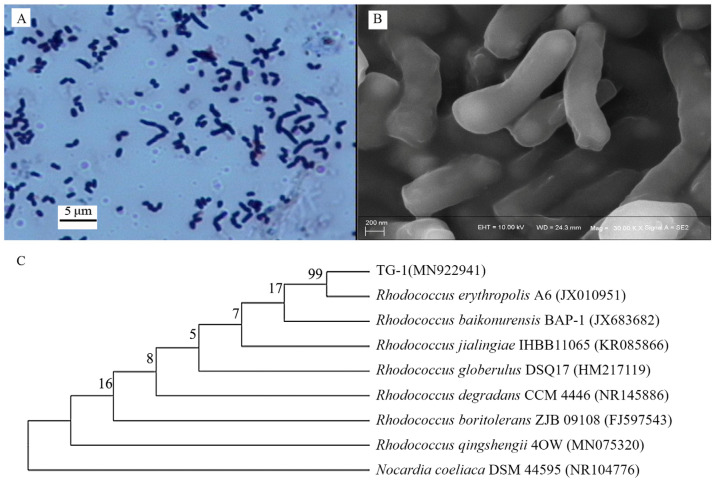
(**A**,**B**): morphological characteristics of strain TG-1. (**C**): phylogenetic tree of strain TG-1 based on 16S rRNA gene sequence.

Strain TG-1 exhibited Gram-positive (Figure 1A), short, rod-like phenotypic characteristics (Figure 1B) without spores. Analysis of the 16S rRNA gene sequence revealed the highest similarity (94%) between strain TG and *Rhodococcus erythropolis* A6 (GenBank accession No. JX010591) (Figure 1C). Hence, the isolated strain was identified as a Rhodococcus strain (GenBank accession No. MN922941).

### 3.2. Effect of Metal Ions on Toluene Degradation

**Figure 2:**
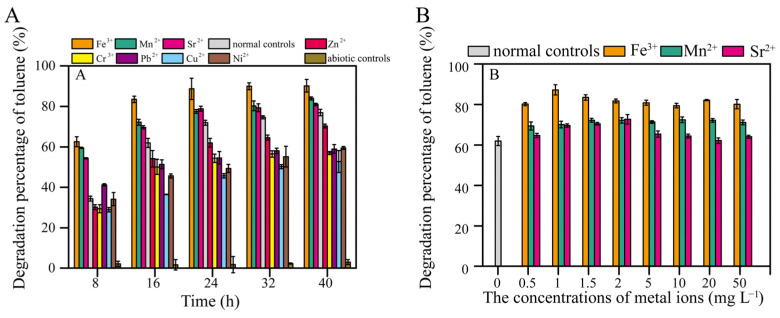
(**A**) Effects of different metal ions (2 mg L^−1^) on toluene degradation. (**B**) Effects of different concentrations of Fe^3+^, Mn^2+^, and Sr^2+^ ions on toluene degradation at 16 h. Note that normal controls were inoculated but lacked metal ions, while chemical controls lacked inoculation but contained metal ions.

As the cultivation time increased, the degradation rate of 600 mg L^−1^ toluene by TG-1 increased from 34.3% (8 h) to 61.9% (16 h). Toluene degradation by TG-1 was obviously increased by Fe^3+^, Mn^2+^, and Sr^2+^. Fe^3+^ showed the greatest promotion effect. Conversely, inhibition of toluene degradation was observed for Zn^2+^, Cr^3+^, Pb^2+^, Cu^2+^, and Ni^2+^ (Figure 2A). Hu et al. reported that Pb^2+^ inhibited growth and changed the surface morphology of the *Rhodococcus* sp. HX-2 [11]. Chen et al. also reported that Cu^2+^ inhibited benzo[a]pyrene biodegradation by *Stenotrophomonas maltophilia* owing to bacterial cell wall damage [17]. These reports suggest that heavy metals may reduce the degradation capacity by compromising cell integrity. Other reports have shown that heavy metals inhibit microorganisms by blocking essential functional groups, interfering with the incorporation of essential metal ions into biological molecules, or directly inhibiting enzyme activity to suppress degradation [18]. When Fe^3+^, Mn^2+^, and Sr^2+^ were added at concentrations of 0.5 mg L^−1^ or higher, toluene degradation by TG-1 was enhanced (Figure 2B). Further experiments with TG-1 found that the greatest positive effects on toluene degradation due to Fe^3+^ occurred even at concentrations as low as 1 mg L^−1^ (the toluene degradation percentage increased from 61.9% to 87.2% at 16 h) (Figure 2B). Therefore, we selected the Fe^3+^ concentration of 1 mg L^−1^ for subsequent experiments to investigate how Fe^3+^ promotes the degradation of toluene by TG-1.

Furthermore, the functional genes of bacteria and the mechanism of the interaction between bacteria and Fe^3+^ both require further clarification. Therefore, transcriptomic analysis was employed to investigate the mechanism of the interaction between strain TG-1 and Fe^3+^ at the genetic level in subsequent experiments.

### 3.3. Characterization of the Interaction Between Strain TG-1 and Fe^3+^

**Figure 3:**
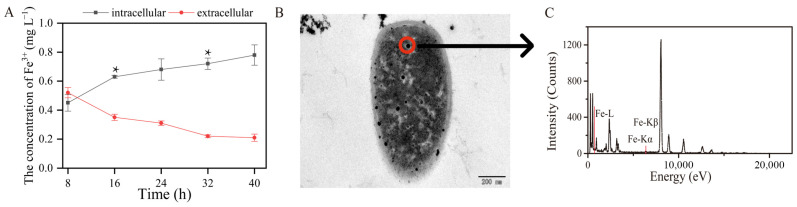
(**A**): the total extracellular and intracellular Fe contents changed over time (* *p* < 0.05). (**B**,**C**): The TEM and EDS of the Fe^3+^ absorption by TG-1.

The initial concentration of Fe^3+^ in this experiment was 1 mg L^−1^. The intracellular and extracellular concentrations of Fe^3+^ were 0.73 and 0.21 mg L^−1^ after 16 h of toluene biodegradation, respectively (Figure 3A). This indicates that during the toluene degradation process, Fe^3+^ was transported into the TG-1 cells. The ability of strain TG-1 to absorb Fe^3+^ was relevant during toluene biodegradation. Increasing the bioavailability of metal ions was critical for biodegradation. These results were the basis for stimulating toluene biodegradation by strain TG-1 using Fe^3+^. In the Fe^3+^-supplemented system, the percentage of toluene degraded by TG-1 increased, showing that Fe^3+^ exerted a positive effect on toluene degradation. Some other studies have also reported that Fe^3+^ can promote the degradation of pollutants. Qi et al. found that Fe^3+^ significantly promoted the growth of *Pseudomonas* T4, then increased the oxytetracycline degradation percentage from 34.1% to 65.3% [19]. In addition, Feng et al. found that Fe^3+^ could also promote aerobic denitrification [20]. The maximum nitrate reduction rate of *Pseudomonas stutzeri* T13 increased from 9.55 mg-N L^−1^ h^−1^ to 19.65 mg-N L^−1^ h^−1^ with Fe^3+^ ion concentrations increasing from 0.004 to 0.036 mmol L^−1^. These studies showed that Fe^3+^ promoting the microbial degradation of pollutants is a common phenomenon. This phenomenon may be due to the addition of Fe^3+^. Therefore, TEM was used to demonstrate that Fe^3+^ was transported into the TG-1. The results showed TG-1 with a normal appearance in the Fe^3+^-supplemented system (Figure 3B). TEM showed that black dots were visible in the TG-1. EDX spectral analysis also indicated that Fe^3+^ was indeed present in the TG-1 (Figure 3C), which may have been due to cellular uptake of Fe^3+^ into the TG-1.

### 3.4. The Activity of C12O

**Figure 4:**
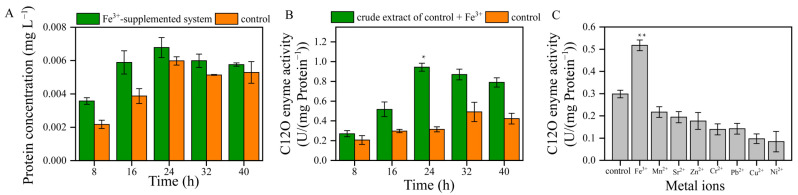
(**A**): the protein concentrations of the Fe^3+^-supplemented system and the control. (**B**): the C12O enzyme activity of the crude extract of the Fe^3+^-supplemented system and the control (* *p* < 0.05). (**C**): the effects of metal ions on the C12O enzyme activity at 16 h (** *p* < 0.01).

The catechol 1, 2-dioxygenase (C12O) was an important enzyme in the degradation pathway of aromatic compounds, and the activity of C12O was key for enhancing the rate at which toluene was degraded by bacteria. The protein concentration of the Fe^3+^-supplemented system was higher than that of the control between 8 and 24 h (Figure 4A). Thus, Fe^3+^ promoted the protein synthesis of strain TG-1.

In the control, the C12O enzyme’s mass-specific activity was between 0.207 and 0.491 U/mg of protein (Figure 4B). The mass-specific activity of the protein in the control was observed between 24 and 32 h, and the enzyme’s mass-specific activity values were 0.315 and 0.491 U/mg of protein, respectively. The structures of other members of the intradiol dioxygenase family, C12O from the Gram-negative bacteria *Acinetobacter* sp. ADP1 and *Pseudomonas arvilla* C-1, were also solved [21]. The tertiary structure of C12O is similar to that found in catechol 3,4-dioxygenase (C34O), although it contains a novel helical zipper motif at the interface of the two subunits and contains two molecules of bound phospholipids. The active site of C12O exhibits the same arrangement of Fe^3+^ ligands found in C34O. The first crystallographic structures of a C12O from a Gram-positive bacterium (*Rhodococcus opacus* 1CP) showed an Fe^3+^-containing enzyme specialized in the aerobic biodegradation of catechol. The structure of the intradiol dioxygenases revealed a trigonal bipyramidal Fe^3+^ site with four endogenous amino acid ligands [22]. Fe^3+^ with phenolate moieties of tyrosine residues plays an important role in the enzyme function and in stabilizing the active site geometries of dioxygenases.

The induction of C12O enzyme activity in the Fe^3+^-supplemented system was different from the trend observed in the control (Figure 4B). The C12O enzyme activity in the Fe^3+^-supplemented system showed a downward trend and was higher than that in the control. At 24 h and 32 h, the C12O enzyme activity of the Fe^3+^-supplemented system was 2.99 and 1.77 times higher than that of the control, confirming that Fe^3+^ stimulated the C12O enzyme activity. Our results are consistent with Liu’s research, which showed that Fe^3+^ could enhance the activity of C12O [15]. A further finding was that other metal ions did not stimulate C12O enzyme activity (Figure 4C). These results further demonstrate that Fe^3+^ has a significant impact on the activity of C12O.

### 3.5. Transcriptome Sequence Assembly and Analysis

**Figure 5:**
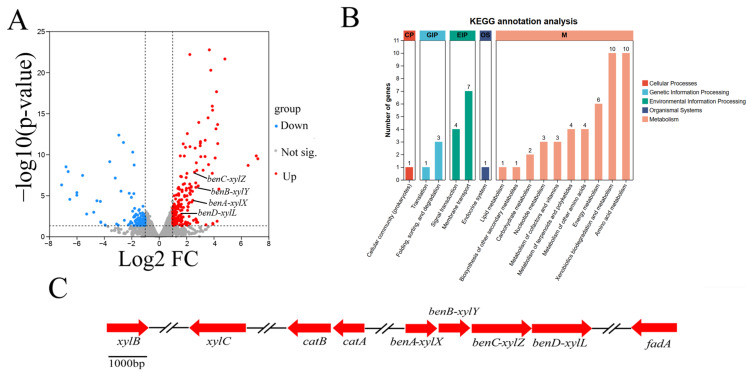
The DEGs of CK vs. T ((**A**): volcano plot; (**B**): KEGG annotation analysis). The gene clusters of toluene degradation (**C**).

In total, 213 up-regulated DEGs and 132 down-regulated DEGs, corresponding to 4.8% of all genes, were predicted for the *Rhodococcus* sp. TG-1. A volcano plot illustrates the expression distribution of the DEGs (Figure 5A). The functions of the DEGs were further classified via the Kyoto Encyclopedia of Genes and Genomes (KEGG). The top enriched pathways included xenobiotics biodegradation and metabolism, amino acid metabolism, energy metabolism, and membrane transport (Figure 5B). KEGG enrichment analysis showed that Fe^3+^ mediated the up-regulation of four genes in the toluene degradation pathway, including *benA-xylX*, *benB-xylY*, *benC-xylZ,* and *benD-xylL*. Specifically, the expression of these four genes in the Fe^3+^-supplemented system was 5.27-, 5,46-, 4.14-, and 2.92-fold higher than without the Fe^3+^ treatment at 16 h, respectively. These genes are similar to the reported toluene degradation genes in *Pseudomonas putida* [23].

### 3.6. Toluene-Degrading Genes

**Figure 6:**
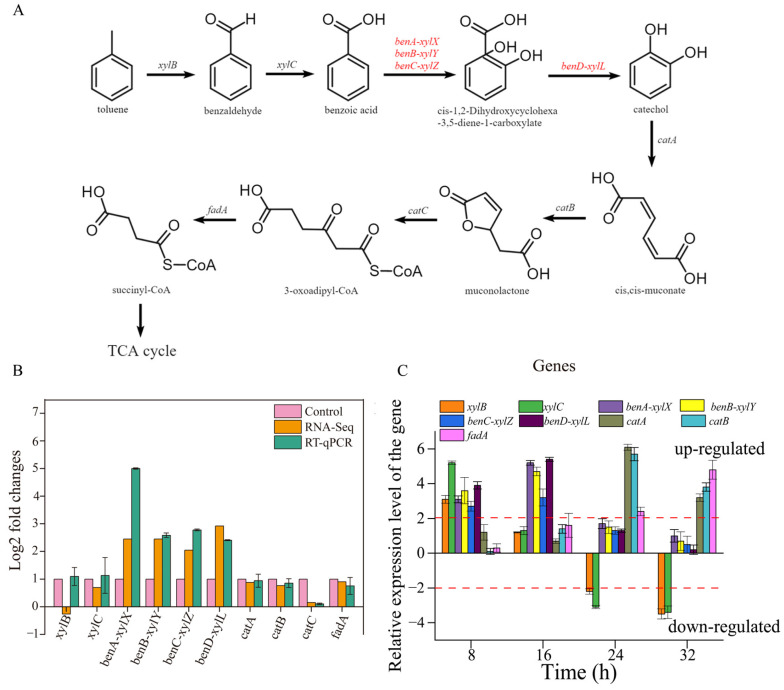
(**A**): the pathway of toluene degradation in TG-1. (**B**): The log2 fold changes of DEGs associated with toluene degradation. (**C**): the expression of toluene degradation genes over time.

The whole-genome sequences of TG-1 substantiate the existence of a metabolic pathway for toluene. Toluene was degraded to benzoic acid by aryl-alcohol dehydrogenase and benzaldehyde dehydrogenase and was further changed into catechol and CO_2_ by benzoate 1,2-dioxygenase, FCD domain-containing protein, and 1,6-dihydroxycyclohexa-2,4-diene-1-carboxylate dehydrogenase. Catechol was converted to 3-oxy-adipate by C12O, muconate cycloisomerase, and muconolactone delta-isomerase, then converted into succinyl-CoA by acetyl-CoA acyltransferase (Figure 6A). Finally, succinyl-CoA was converted via the citrate cycle, and the pathway was the same as in previous reports [3].

The expression of *benA-xylX*, *benB-xylY*, *benC-xylZ*, and *benD-xylL,* encoding the large benzoate 1,2 dioxygenase subunit (*benA-xylX*), the small benzoate 1,2-dioxygenase subunit (*benB-xylY*), FCD domain-containing protein (*benC-xylZ*) and 1,6-dihydroxycyclohexa-2,4-diene-1-carboxylate dehydrogenase (*benD-xylL*), enabled TG-1 to initiate toluene degradation. The transcriptome analysis showed that Fe^3+^ significantly enhanced the expression of these four genes when compared with the control (without Fe^3+^). This result suggested that Fe^3+^ played a role in promoting the expression of these four genes in TG-1. This inference was supported by the result that the TG-1 in the Fe^3+^-supplemented system exhibited an excellent toluene degradation ability compared to that in the control treatments (without Fe^3+^) 16 h into the experimental period. Meanwhile, other genes related to toluene degradation were not up-regulated or down-regulated (Figure 6B). A previous study reported that gene expression for dioxygenase in the benzoate degradation pathway increased over time due to a separate pathway. The expression levels of ten genes related to toluene degradation by TG-1 in the Fe^3+^-supplemented system were studied by qPCR. The results are expressed as the relative normalized expression of the ten genes of TG-1 in the Fe^3+^-supplemented system compared with the TG-1 in the control treatments (without Fe^3+^). The results indicate that *xylB* and *xylC* were up-regulated by 3.1- and 5.2-fold at 8 h (Figure 6C). *xylB* and *xylC* were not up-regulated or down-regulated until 24 h, as the degradation proceeded. *xylB* and *xylC* were down-regulated at 24 h and 32 h, respectively. *benA-xylX*, *benB-xylY*, *benC-xylZ,* and *benD-xylL* were up-regulated by 3.1-, 3.6-, 2.7-, and 3.9-fold at 8 h, and these four genes were up-regulated by 5.2-, 4,7-, 3.2-, and 5.4-fold at 16 h. Then, they were not up-regulated or down-regulated from 24 to 32 h. *catA* and *catB* were up-regulated by 6.1- and 5.7-fold at 24 h and by 3.2- and 3.8-fold at 32 h. *fadA* was up-regulated of 2.4-fold at 24 h and 4.8-fold at 32 h. In the Fe^3+^-supplemented system, appropriate changes in gene expression quickly followed. At 8 h, *xylB* and *xylC* were up-regulated, which illustrated that Fe^3+^ enhanced the biosynthesis of xylulokinase and benzaldehyde dehydrogenase. Xylulokinase and benzaldehyde dehydrogenase could accelerate the process of toluene degradation into methyl benzoate in TG-1. Then, more methyl benzoate could stimulate the biosynthesis of the large benzoate 1,2-dioxygenase subunit (*benA-xylX*), the small benzoate 1,2-dioxygenase subunit (*benB-xylY*), FCD domain-containing protein (*benC-xylZ*), and 1,6-dihydroxycyclohexa-2,4-diene-1-carboxylate dehydrogenase (*benD-xylL*), so the expression of these four genes was up-regulated at 16 h. Analogously, subsequent metabolic genes were up-regulated over time.

## 4. Conclusions

In this study, a toluene-degrading bacterium was isolated, identified, and named *Rhodococcus* sp. TG-1. The results of GC showed that TG-1 degraded 600 mg L^−1^ toluene by 61.9% within 16 h. Some metal ions were found to enhance the efficiency with which toluene was degraded by TG-1, and Fe^3+^ showed the greatest promotion effect. After adding 1 mg L^−1^ Fe^3+^ to the culture medium, the toluene degradation percentage increased from 61.9% to 87.2% at 16 h. Through TEM and EDS analysis, we confirmed that TG-1 indeed transported Fe^3+^ into its cells during toluene degradation.

The catechol 1,2-dioxygenase (C12O) is an important enzyme in the degradation pathway of aromatic compounds, and Fe^3+^ makes up the active site of C12O. The experimental results show that the addition of Fe^3+^ significantly enhanced the activity of C12O. The mechanism by which Fe^3+^ promotes toluene degradation was analyzed at the genetic level through transcriptomic analysis and RT-qPCR analysis. The experimental results show that genes related to toluene degradation were up-regulated after the addition of 1 mg L^−1^ Fe^3+^. benA-xylX, benB-xylY, benC-xylZ, and benD-xylL were up-regulated by 5.2-, 4,7-, 3.2-, and 5.4-fold at 16 h. These results indicate that Fe^3+^ promotes toluene degradation in two ways: by enhancing the expression of degradation-related genes and by increasing the activity of C12O. The mechanism by which Fe^3+^ ions promote toluene degradation will provide reference value for subsequent bioremediation applications of strain TG-1.

## Figures and Tables

**Table 1:** Primer design.

Gene Name	Primer	Sequences
*xylB*	xylB-F	CGAAGTCGGTGCTACCGGAA
	xylB-R	TGCGTGCGCCAGAGCTGTTG
*xylC*	xylC-F	TCGCTCATCAGCAATGGCAC
	xylC-R	CTGCCTTACGGATCACCGAA
*benA-xylX*	benA-xylX-F	ATGACGGATATGTTGGATGC
	benA-xylX-R	GGTGAAGTAGTCGCCGACGT
*benB-xylY*	benB-xylY-F	ACCAATGCCGTCGCCCTCGA
	benB-xylY-R	GATTCGGAACACGCGGTCCT
*benC-xylZ*	benC-xylZ-F	ATTCCGCTCGACTGCCGCGA
	benC-xylZ-R	GGTGGACGTGTACGAACCGG
*benD-Xyl*	benD-Xyl-F	CACGAGATTCTTCGCCGGCC
	benD-Xyl-R	TGCGTGCGCCAGAGCTGTTG
*catA*	catA-F	AGCCCCACCGCAGTAGGTTC
	catA-R	CCGAACTGGCCGATGTCGAT
*catB*	catB-F	CCTGTCGATCGTCTCCATCG
	catB-R	CGGGAGCGATGTACTTCTCG
*fadA*	fadA-F	GTCATCGTCGACGCAGTACG
	fadA-R	CGGCAGCGAGTGCCGCTGTG

## Data Availability

The original contributions presented in this study are included in the article. Further inquiries can be directed to the corresponding authors.
